# High-Performance Pressure Sensor for Monitoring Mechanical Vibration and Air Pressure

**DOI:** 10.3390/polym10060587

**Published:** 2018-05-27

**Authors:** Yancheng Meng, Hongwei Li, Kunjie Wu, Suna Zhang, Liqiang Li

**Affiliations:** 1 School of Nano-Technology and Nano-Bionics, University of Science and Technology of China, Hefei 230026, China; ycmeng2015@sinano.ac.cn; 2 Suzhou Institute of Nano-Tech and Nano-Bionics (SINANO), Chinese Academy of Sciences, Suzhou 215123, China; hwli2015@sinano.ac.cn (H.L.); kjwu2014@sinano.ac.cn (K.W.); snzhang2014@sinano.ac.cn (S.Z.)

**Keywords:** pressure sensor, ultra-fast response, mechanical vibration and air pressure

## Abstract

To realize the practical applications of flexible pressure sensors, the high performance (sensitivity and response time) as well as more functionalities are highly desired. In this work, we fabricated a piezoresistive pressure sensor based on the micro-structured composites films of multi-walled carbon nanotubes (MWCNTs) and poly (dimethylsiloxane) (PDMS). In addition, we establish efficient strategies to improve key performance of our pressure sensor. Its sensitivity is improved up to 474.13 kPa^−1^ by minimizing pressure independent resistance of sensor, and response time is shorten as small as 2 μs by enhancing the elastic modulus of polymer elastomer. Benefiting from the high performance, the functionalities of sensors are successfully extended to the accurate detection of high frequency mechanical vibration (~300 Hz) and large range of air pressure (6–101 kPa), both of which are not achieved before.

## 1. Introduction

Pressure sensor is one of key components of flexible and wearable electronic products [1,2,3,4,5,6,7,8,9,10,11,12,13,14]. For realizing practical applications, the high performance (especially the high sensitivity and fast response time) and diverse functionalities (i.e., capacity for detecting more kinds of stimulus) are highly desired [15,16]. However, the sensitivities of current pressure sensors are generally in the range from several to one hundred of kPa^−1^ [15,17,18,19,20,21], and the response times are at the level of milliseconds or larger [16,20,22,23,24,25]. Specially, the piezoresistive pressure sensor, which is operated via changing the contact area and thus the resistance to tune the output current, have great potential in flexible systems, while their sensitivity are still in very low levels [10,12,25]. For example, Choong et al. reported a classical piezoresistive pressure sensor that is based on the conductive polymer coated cone-shape geometry, in which the sensitivity is only 10.32 kPa^−1^. Until now, no efficient strategy is developed to greatly improve the performance of piezoresistive pressure sensor far beyond the current level. In addition, regarding the functionalities (application fields) of pressure sensors, most of them aimed to monitor or reflect the mechanical state of human body or robot [16,26,27,28,29,30,31]. While there are rare applications in detecting the mechanical quantity in the environment such as high frequency (over hundreds of Hz) mechanical vibration and air pressure, both of which are closely related to human life. Epidemiological and experimental evidences suggest that if human is exposed to vibration at frequencies higher than 100 Hz, the risk of injury dramatically increases [32]. Conventionally, the microelectromechanical systems (MEMS) were proposed to detect the vibrations, while these devices are intrinsically rigid, which are unfavorable to realize flexible electronics applications. Recently, numerous flexible sensors such as pressure sensor and strain sensor were proposed to detect the classical mechanical quantities [22,23,24,25]. However, they have very limited performance to detect vibrations, especially in high frequency region. To realize the detection of high frequency vibration, fast response should be achieved which is difficult because the polymer elastomers employed in these sensors have a poor response to high frequency excitations for their viscoelasticity and poor mechanical recovery.

Air pressure is also an important environmental factor in the daily life, especially for someone exposed to positive or negative pressure constantly. The traditional air pressure gauge is rigid and cumbersome. Optical fiber-based air pressure sensors were fabricated with compact size in recent years [33,34], while a complex test system is needed for signal detection which may prevent these devices from being used in flexible electronics at present. Therefore, it is a necessary to propose another light, flexible, wearable, easily detectable air pressure sensor.

According to the working principle, piezoresistive pressure sensor has potential for realizing the detection of high frequency vibration and air pressure. Meanwhile, the air pressure gauge based on flexible pressure sensor is not reported until now. The limited functionalities might stem from the relatively low performance as well as the lack of proper strategy for the device fabrication.

In this article, we present a simple yet efficient strategies to improve the performance of our sensor to far beyond the current level of the piezoresistive pressure sensors. The sensitivity is improved to be a high level of about 474 kPa^−1^ and response/relaxation time is shorten as small as 2 μs/74 μs. Meanwhile, the sensors show large range of pressure sensing from 0 Pa to 110 kPa and a low detection limit of 0.6 Pa. Benefiting from these outstanding properties, we prepare vibration sensor and air presser sensor based on our pressure sensor, which can be used to detect the vibration in the frequency range of 1 to ~300 Hz as well as air pressure from 6 to 110 kPa.

## 2. Materials and Methods

### 2.1. Material Synthesis

Trichloromethane was used to dissolve the poly (dimethylsiloxane) (PDMS) and allowed dispersion of multi-walled carbon nanotubes (MWCNTs). 2 wt % was chosen as the quality ratio of MWCNTs (purchased from Shichuang Company, Changzhou, China; outer diameter: 15–20 nm) to PDMS for all of our samples. To start mixing, the PDMS base polymer, dry MWCNTs and Trichloromethane were added into beaker successively. Then the beaker was positioned on top of heat source with the temperature 80 °C, and the mixture was continual stirred for 4 h to promote MWCNTs dispersing into PDMS and trichloromethane evaporating from the mixture. After trichloromethane reached full evaporation, the PDMS curing agent (1:10 weight ratio to base polymer) was added into the mixture and stirred for 5 min. Then the conductive materials were readied and waited to spin-coating as thin layer.

### 2.2. Micro-Structured Composite Films Fabrication

Silicon slice was chosen as mold which have recessed similar-pyramid structures, which was acquired from Shichuang Company, Changzhou, China. The surface of the silicon mold was coated with OTS (octadecyltrichlorosilane) to avoid the adhesion of the composite film. Then used the spin-coating method to prepare the micro-structured composite films, after degassing and curing in the vacuum chamber with 100 °C temperature and 100 Pa vacuum level, the micro-structured composite layers were peeled off.

### 2.3. Signal Detection System

Keithley 4200 was used to apply the voltage into the sensor. When constant voltage is applied to the sensor, the current will flow through the apex of pyramid-like microstructure to the indium tin oxide poly (ethyleneterephthalate) (ITO-PET) layer. The sensor is operated via changing the contact area and thus the resistance under varying pressures. We successively applied a series of step-like pressures to the sensor, and then released the pressure in reverse sequence. At the same time, the output current can be recorded by the Keithley 4200 in situ under various pressure condition.

### 2.4. Vibrations Calibration

The commercial standard vibration sensor should be fixed on the mechanical vibration source. It can monitor the vibration in situ. When the mechanical vibration source under operating, vibration’s accelerations and frequencies can be measured by standard sensor and recorded in computer.

### 2.5. Air Pressure Sensor Fabrication

For the first step, the high-purity paraffin was prepared as adhesive. Second, we put the ITO-PET layer on oven and wait the temperature upper to 90 °C, then paint high-purity paraffin on the ITO-PET layer with a closed line frame and put the indium tin oxide (ITO)-coated flexible poly (ethyleneterephthalate) (PET) layer off oven, waiting it cool to room temperature. The third step is that the conductive layer was stacked onto ITO-PET layer matched the line frame of high-purity paraffin and heat the device for the temperature upper to 75 °C under different vacuum degree. Two minutes later turn off the heater and wait the device cooling to room temperature naturally. Finally, ITO-PET layer and conductive layer pasted together along the closed paraffin line frame automatically, and the vacuum level was hold between two layers as same as the vacuum degree in packaging condition.

## 3. Results and Discussion

### 3.1. Sensor Fabrication and Working Principle

**Figure 1:**
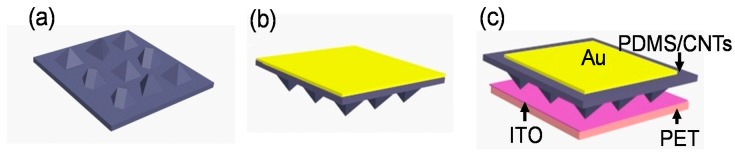
Schematic illustration of sensor fabrication. (**a**) Conductive layers with micro-structures are cured and peeled off from Si mold; (**b**) Au was deposited on the reverse side of conductive layers; (**c**) A sensor unit was constructed through a sandwich structure comprising the micro-structured conductivity layers and indium tin oxide (ITO)-coated poly (ethyleneterephthalate) (PET) film.

Figure 1 schematically illustrated the fabricated process of our sensor. The former conductive layer was prepared by doping MWCNTs into PDMS, which has pyramid-like patterns (Figure 1a), being the replica of that on the recessed pyramid-like silicon mold. MWCNTs were used as filler materials because of their exceptional mechanical, electrical, thermal, and magnetic properties coupled with very large interfacial contact area [35,36]. At the same time, the blending of MWCNTs may enhance the elastic modulus of PDMS films. This property may render the micro-structured flexible films to have fast recovery progress and avoid wrinkling under operation in ultra-high pressure range as demonstrated in the contents below.

Au was deposited on the reverse side of conductive layers based on the vacuum evaporation coating method (Figure 1b). Subsequently, the micro-structured conductivity layer is affixed to a flat conductive ITO-PET film face-to-face, which has a sandwich structure (Figure 1c).

**Figure 2:**
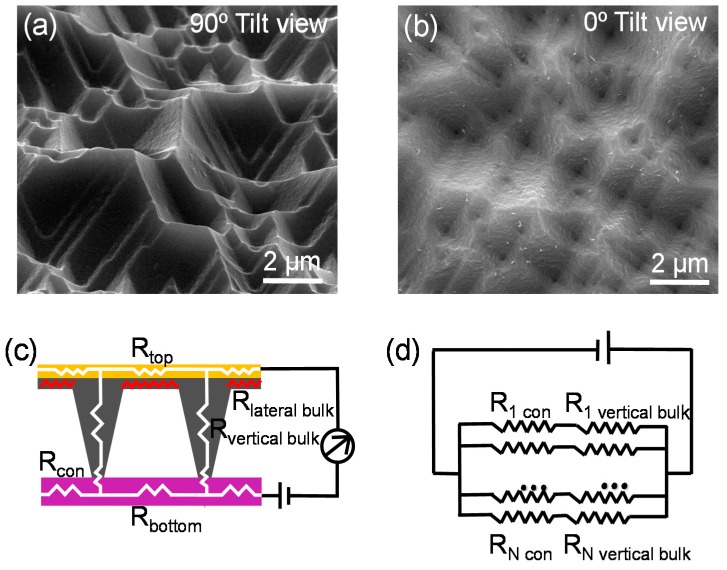
SEM images of conductive layer with pyramid-like patterns (**a**) with 90° tilt view and (**b**) with 0° tilt view. (**c**) A simple circuit model corresponding to random individual pyramid-like microstructure. The pressure independent lateral bulk transporting resistance (*R*_lateralbulk_, red folding line) is replaced by top electrode resistance (*R*_top_). (**d**) The proposed equivalent circuit diagram of entire sensor.

The sensor is consists of two conductive layers: one is poly (ethyleneterephthalate) (PET) substrate coated with indium tin oxide (ITO), and the other is micro-structured composite films with the uniform thickness of about 100 μm. It has pyramid-like patterns (Figure 2a,b) on one side replicated from the silicon mold. The largest height of these patterns is approximately uniform, and the value is about 4 μm. On the other side, one high conductive Au film with controlled area was deposited as top conductive electrode.

The Au film is highly important for the sensitivity improvement due to the following reasons: The total resistance of pressure sensor mainly consists of contact resistance (*R*_con_) and bulk resistance (*R*_bulk_), so it would be favorable to achieve high sensitivity if *R*_con_ and *R*_bulk_ may change with pressure simultaneously. Generally, *R*_con_ strongly depends on the pressure, but *R*_bulk_ shows weak or no dependence on the pressure in some pressure sensors with large spacing distance (several mm to cm) between two high conductive electrodes, which might be one of main reasons for the limited sensitivity. To overcome this problem, minimization or elimination of *R*_bulk_ would be an effective way. In our work, the lateral bulk transporting resistance (*R*_lateralbulk_) of the top conductive layer is replaced by the negligible Au electrode resistance (*R*_top_) as Figure 2c illustrated, which successfully eliminates the lateral bulk resistance that shows weak dependence on pressure. Therefore, the total resistance of individual of pyramid-like microstructure is sum of vertical bulk resistance and contact resistance (Figure 1c), both of which are highly dependent on the pressure as demonstrated and explained in the contents below. At the same time, each pyramid-like microstructure is connected in parallel mode (Figure 2d), so the total resistance of the sensor is highly depended on the pressure too. This fact means the total resistance of the sensor device can be effectively tuned by the changes of pressure, which may enhance the sensitivity significantly. When constant voltage is applied to the sensor by Keithley 4200, the current will flow through the apex of pyramid-like microstructure to the ITO-PET layer. The sensor is operated via changing the contact area and thus the resistance under varying pressures.

### 3.2. High Sensitivity and Theoretical Analysis

**Figure 3:**
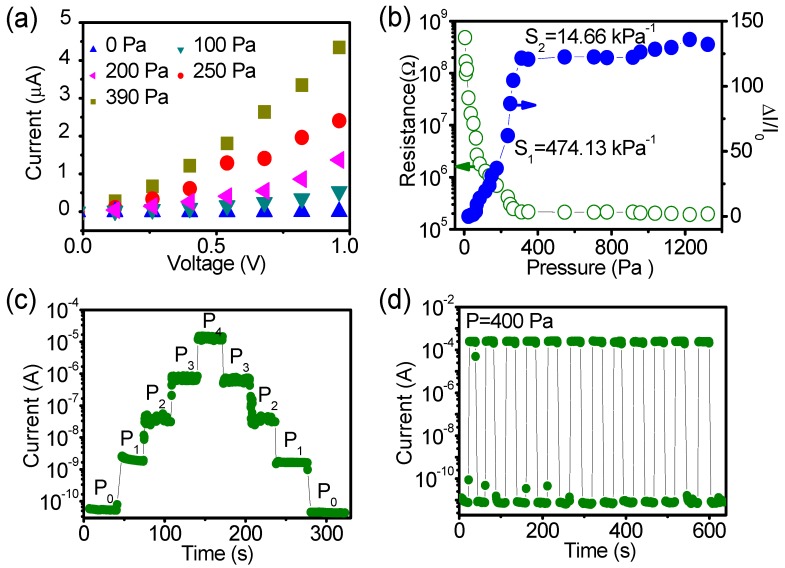
Sensitivity and repeatability of the sensor with large area of Au film (4.4 × 10^−5^ m^2^). (**a**) Current-voltage curves of the device under different amount of pressure loading; (**b**) The dependency of sensitivity and total resistance on pressure under the same pressure range (0 to 1400 Pa); (**c**) Current response of the sensor under step pressure and certain applied voltage of 5 V, *P*_0_ = 0 Pa, *P*_1_ = 11 Pa, *P*_2_ = 25 Pa, *P*_3_ = 52 Pa and *P*_4_ = 118 Pa; (**d**) Cycle test of sensor under pressure of 400 Pa and voltage of 1 V.

The electrical properties of the pressure sensor with Au film area of 4.4 × 10^−5^ m^2^ (larger area) were measured under variable loads. From current-voltage curves (Figure 3a), it can be seen that the sensor’s resistance exhibits an obvious decreases as the applied pressure increases. Under the pressure of 0 Pa, the current reaches to 3.79 × 10^−8^ A at the voltage of 1 V, which is set as the initial current.

Sensitivity is the most important parameter of pressure sensor, because it determines the measurement accuracy and effectiveness of the device [23]. In order to calculate the sensitivity of our sensor, an elaborate measurement was performed for the pressure increasing from 0 to 1400 Pa. The sensors showed maximum resistance without loading. The application of pressure leaded to a sharp decline of the total resistance of the device until an inflection point appeared at 400 Pa, and then this tendency approached saturation with applying higher pressures (Figure 3b). The different dependencies of resistance on pressure enable the curve of relative variation of current (Δ*I/I*_0_) versus pressure to show distinct tendency with two regions. Near linear region (0 to 400 Pa) shows the sensitivity up to 474.13 kPa^−1^ and approximate saturated region (400 to 1400 Pa) shows sensitivity of 14.66 kPa^−1^, respectively, which are plotted as blue curve in Figure 3b. The sensitivity in low pressure regime is quite high compared with the reported values of piezoresistive pressure sensors calculated with the standard method [10,12,25]. Then a similar test was performed in the large pressure range from 5 to 110 kPa, and the sensitivity of 10.46 kPa^−1^ was got as shown in Figure S1. These results indicate that our sensors have excellent performance at low pressure region and larger pressure measurement range.

To further testify the high sensitivity and repeatability of our sensor in low pressure regime, we successively applied a series of step-like pressures with 0, 11, 25, 52, 118 Pa to the sensor, and then released the pressure in reverse sequence. The current changed without hysteresis, and retained nearly the same level for the certain pressure during the loading and unloading the pressure (Figure 3c and Figure S2). Furthermore, the current changed over several orders of magnitude at the applied pressure of 118 Pa, confirming the high sensitivity. At same time, another repeatability tests were performed under pressure switching between 0 to 400 Pa (Figure 3d) and 0 to 14 Pa (Figure S3), respectively, all of which confirmed the high repeatability.

The above experimental results show that our sensor produces a high sensitivity (about 480 kPa^−1^), which is higher than most of reported results. To interpret this performance, a mathematical calculation and analysis are performed. For simplicity purposes, the pyramid-like microstructure was approximated as cone in the process of calculation. Based on Holm’s theory and steady electric field approximation [37,38], contact and vertical bulk resistant can be calculated (Supporting Discussion S1, Figure S4), respectively, and the total resistance of sensor can be expressed as Equation (1)

(1) $$R_{total}=\frac{1}{N}\cdot R_{n\;total}=\frac{1}{N}\cdot \left(C\cdot \frac{1}{r_{1n}}+D\cdot \frac{1}{r_{2n}}\right)$$

*r*_1n_ is radius of cone’s section deformed under pressure, *r*_2n_ is radius of cone’s back surface that is a constant (schematically illustrates at Figure S5). *C* and *D* are constants, which are abbreviations of the product of several constants (Supporting Discussion S1). *N* is number of pyramid-like microstructure below the Au film.

For a certain sensor, *N*, *C*, *D* and *r*_2n_ are constants, so *R*_total_ is proportional to 1/*r*_1n_. With the increasing of pressure, 1/*r*_1n_ will decrease, rendering *R*_total_ to reduce continuously. In the other words, the sensor’s total resistance is highly dependent on the pressure changes.

Based on Equation (1), we have following relation

(2) $$\frac{dR_{total}}{dr_{1n}}=-\frac{C}{N}\cdot r_{1n}^{-2}$$

That is, the slope of Equation (1) is −1/*r*^2^_1n_, and its absolute value decreases with the increasing of *r*_1n_. According to the basic principle of power equation, Equations (1) and (2) indicate that with the increasing of pressure, *R*_total_ will decrease continuously with gradually weakened tendency, until approaching to a constant (*r*_1n_ will approach to *r*_2n_ under enough pressure), which is in good accordance with the experimental result (Figure 3b).

In fact, the pressure sensor with micro-pyramid structure generally shows nonlinear dependency of resistance or current on the applied pressure, and the sensitivity generally exhibits a reduction in the high-pressure regime [1,9,16]. However, until now this phenomenon is still not well-understood in a rational mode. Equations (1) and (2) provide a mathematical and precise explanation for the nonlinear sensing curve for the first time.

Generally, the sensitivity of pressure sensor is defined as the following relation

(3) $$S=\frac{I-I_{0}}{I_{0}\cdot \Delta P}=\frac{R_{0}}{\Delta P}\cdot \frac{1}{R_{total}}-\frac{1}{\Delta P}$$

*I*_0_/*R*_0_ and *I/R*_total_ are current/total resistance of the sensor without and with load, respectively. Δ*P* is magnitude of pressure changes. Obviously, *S* ∝ (1*/R*_total_), indicating the sensor’s total resistance is key parameter to optimize its sensitivity.

**Figure 4:**
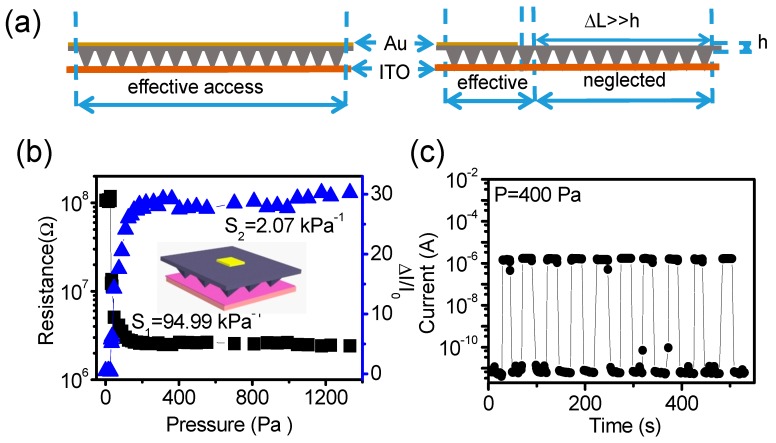
(**a**) Schematic illustration of the difference of the effective access for the sensor with different areas of Au film. The performance of sensor with smaller area of Au film (1 × 10^−6^ m^2^); (**b**) the dependency of sensitivity and total resistance on pressure, inset is the schematic illustration of the sensor with smaller area of Au film; (**c**) relaxation and response curve under pressure switching between 0 and 400 Pa.

As Equation (3) shown, the sensor’s sensitivity is proportional to 1*/R*_total_, and 1*/R*_total_ is proportional to the value of *N* (Equations (1) and (2)). So we can improve the sensor’s sensitivity through increasing the value of *N*. Our sensor consists of conductive top layer and bottom layer. The top layer have vertical resistance (*R*_h_) originating from layer’s thickness (*h* = 100 μm) and lateral resistance (*R*_Δ*L*_) originating from the displacement (Δ*L*) in layer, and the sum of them are bulk resistance of top layer (or the sensor). If depositing Au on the entire opposite side of top layer (Figure 1b), it will have a uniform electric potential under the certain voltage, and *R_ΔL_* can be ignored. This indicates that all of the pyramid-like microstructures below the Au film are effective conductive access schematically illustrated as Figure 4a. If the area of deposited Au film is smaller than the surface area of top layer (Figure 4b inset), *R*_Δ*L*_ originated from the region near outside the Au film cannot be ignored. When the length of displacement (Δ*L*) away from Au film edge is much larger than layer’s thickness (100 μm), *R*_Δ*L*_ will be much larger than *R*_h_ too. It indicates that the corresponding conductive access far away from the Au film edge can be neglected (Figure 4a, right side). So the effective area of electric transmission approximates to the area of Au film, and the values of *N* (conductive access) can be approximated to the number of pyramid-like microstructures below the area of Au film. It thus implies that the value of *N* can be easily modulated by changing the area of Au film.

Therefor the ratio of total resistance can be expressed as the Equation (4) for two sensors with same size and different Au film areas.

(4) $$\frac{R_{\mathrm{total}}^{\prime }}{R_{\mathrm{total}}}=\frac{N}{n}\approx \frac{A}{a}$$

*A*, *a* and *N*, *n* are the area of Au films and the number of pyramid-like microstructure below the Au films for two different sensors, respectively.

Based on Equations (3) and (4), under the same magnitude of pressure change, the ratio of sensitivity can be expressed as Equation (5) for two sensors that with same size and different Au film areas.

(5) $$\frac{S}{S^{\prime }}\approx \frac{I_{0}^{\prime }\cdot V}{I_{0}\cdot V^{\prime }}\cdot \frac{A}{a}$$

It indicates that under the known values of initial current (*I*_0_) and voltage (*V*), the difference of sensitivity of two sensors is only relate to the area of Au film.

To verify the above analyses, we prepared a similar sensor with a smaller area of Au film (1 × 10^−6^ m^2^, Figure 4b inset). Its sensitivity decreased obviously to only 94.99 kPa^−1^ at low pressure region (Figure 4b), which is 5 times less than that of sensor with larger area (4.4 × 10^−5^ m^2^) of Au film (Figure 3b). The ratio is close to the theoretical prediction (8.8 times). It demonstrates the reliability of our theoretical model. On the other hand, under the same cycle test process switching pressure between 0 and 400 Pa, the current changes of sensor with larger area of Au film (Figure 3d) is two orders of magnitude larger than that of the sensor with smaller area of Au film (Figure 4c).

According to the above experimental results and theoretical analyses, one strategy for improving sensitivity can be deduced as follow: the lateral bulk transporting resistance, that is generally independent on the applied pressure, can be eliminated through simply maximizing the area of conductive electrode (Au film). This method may enhance the dependency of sensor’s resistance on the variable contact area caused by the applied pressure, and thus enhance sensor’s sensitivity spontaneously.

### 3.3. Ultra-fast Response Time and Interpretation

**Figure 5:**
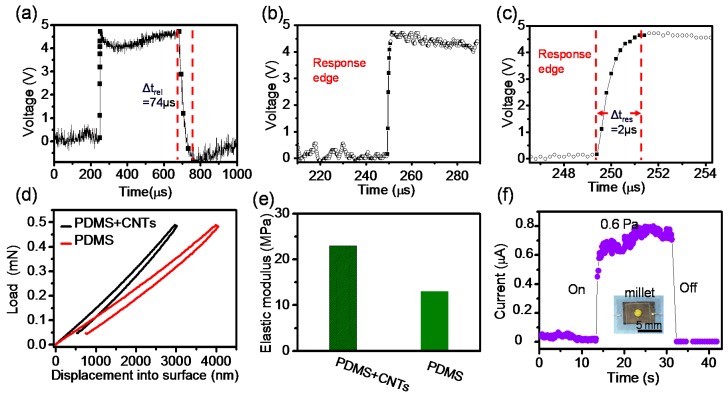
(**a**) The response of our sensor for a mechanical vibration; (**b**) Magnified sensor responses extracted from Figure 3a; (**c**) the further magnified responsive edge, which show the response time is 2 μs. Nano-indentation was used to characterize pure poly (dimethylsiloxane) (PDMS) and composite PDMS/carbon nanotubes (CNT) films; (**d**) The different indentation depths of composite films and pure PDMS films under the same load; (**e**) Elastic modulus of flexible films; (**f**) The current changes with the application and removal of a millet (0.6 Pa) under applied voltage of 5 V.

Another important requirement for high-performance pressure sensor is fast response time [39]. However, the response time of the reported flexible pressure sensors are generally at the level of milliseconds or larger [12,16,18,19,20,21], which may limit their applications in the ultra-fast and high frequency detection. The response time of our sensor was tested under ultrafast stimulus. The signals of mechanical vibration, produced by a mixer operating in high speed, can be detected by our sensor and recorded through the triggered mode of oscilloscope (Figure 5a). In order to calculate the response time, we magnified the responsive edge shown as Figure 5b,c. From the sensing curve, we confirmed the response time and relaxation time are about 2 and 74 μs, respectively, which represent the fastest level so far. To quantitatively understand the improved response characteristics, Nano-indentation was used to characterize elastic properties of pure PDMS and composite PDMS/CNT (Figure 5d). The elastic modulus of pure PDMS films is 13 MPa, which is consistent with the previous studies [40,41]. In contrast, the corresponding value of composite PDMS/CNT layer are 23 MPa, which is nearly two times larger than that of pure PDMS (Figure 5e). It is primary cause for ultra-fast response of our sensors. These results indicate that the response time of flexible pressure sensor can be shortened through enhancing the elastic modulus of polymer elastomers.

On the other hand, limit of detection is another important parameter of sensor too. To test it, a millet with a weight of 3 mg corresponding to a tiny pressure of 0.6 Pa was loaded on and released from the sensor, which resulted in a remarkable change of output current value (Figure 5f).

### 3.4. Typical FunctionalitiesDemonstration

**Figure 6:**
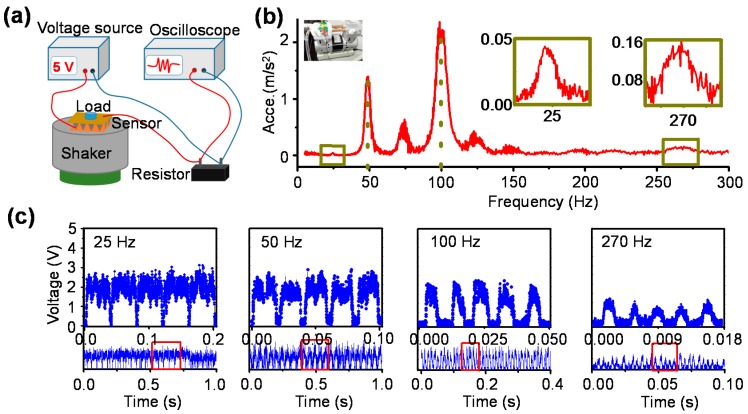
Mechanical vibrations detection. (**a**) The schematic illustration of mechanical vibration measurement platform. The vibrations provided by mechanical pump; (**b**) the relationship between accelerations and frequencies, which was calibrated by standard vibration sensor; (**c**) the typical vibrations with frequencies of 25, 50, 100 and 270 Hz were detected by our sensor.

Our pressure sensors have ultra-fast response time, low detection limit and high sensitivity. They can be used to detect vibrations with various frequencies. Similar to the application of previously reported pressure sensors, the human pulse waveform was monitored firstly (Figure S6). Then the sensors were fixed on different mechanical vibration sources to detect the corresponding vibrations (Figure 6a). Under operation, a mechanical pump can provide a series of vibrations with different frequencies. These vibrations were calibrated by commercial vibration sensor, and the relationship between accelerations and frequencies is shown as Figure 6b. Our pressure sensors can routinely measure the intense vibrations at 50 and 100 Hz (Figure 6c, Video S1). Simultaneously, the weak vibrations at 25 and 270 Hz can also be detected (Figure 6c), indicating the high performance. In fact, our sensor with high sensitivity holds promise for detection higher frequency, which could however be realized due to the lack of reliable high-frequency vibration generator.

To test the generality of our sensor for mechanical vibrations detection, the variable-frequency vibrations below hundreds of Hz provided by mixer under varying operating speeds were monitored by our sensor (Video S2). At same time, the vibrations with the fixed-frequency of 50 Hz provided by another pump, 25 Hz (Video S3) and 220 Hz provided by mixer under different operating speed were detected (Figure S7a–c). In addition, we show the real-time *V*-*t* curves of 1000 times stimulus based on 50 Hz vibration (Figure S7d). The sensor maintain good performance after extremely fast multi-times stimulus, which confirmed the good repeatability of the sensor.

**Figure 7:**
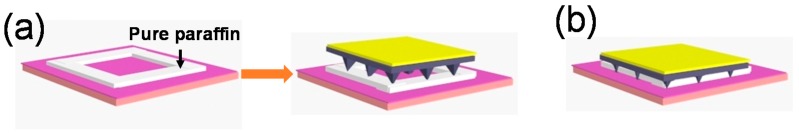
Schematic illustration of the air pressure sensors fabrication, (**a**) high-purity paraffin was painted on the ITO-PET layer with a closed line frame, and the conductive layer was laminated onto ITO-PET layer; (**b**) Two layers are sealed through annealing under various vacuum.

On the other hand, our sensors have large pressure measurement rang (0 Pa to 110 kPa) coupled with the useful sensitivity (14.66 kPa^−1^) in the high pressure region. Meanwhile, the higher elastic modulus of the conductive film may help them to avoid wrinkling under operating in high pressure range. There for they can be used to fabricate flexible air pressure sensors. We propose a packing method that seals an amount of gas between two layers of the sensor to complete the fabrication of air pressure sensor schematically illustrated as Figure 7.

**Figure 8:**
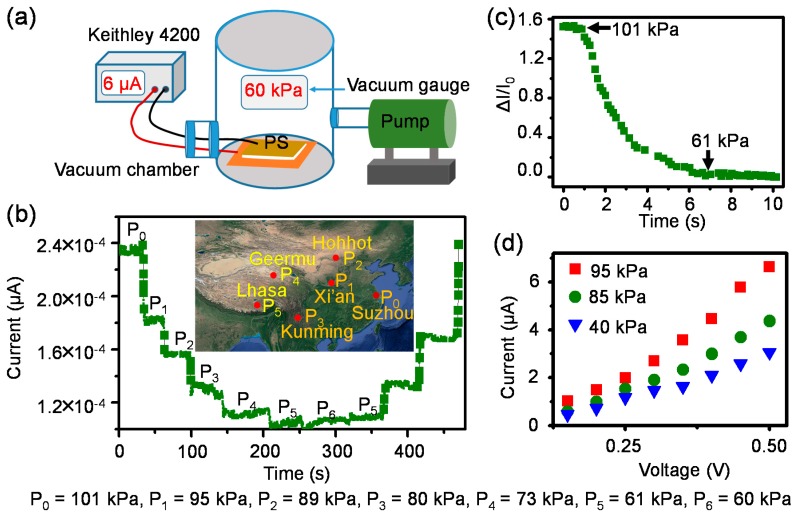
Air pressure detection. (**a**) The schematic illustration of air pressure control and signal record system. The response of device packed under atmosphere for the air pressure changes; (**b**) real-time output current under different air pressure levels; (**c**) Current response under the vacuum level continuously changed from 101 to 60 kPa; (**d**) current-voltage curves under extremely high pressures.

To measure the functionality of packaged sensors (PS) for detecting air pressure changes, an elaborate air pressure control and signal record system was built as schematically illustrated in Figure 8a. Real-time air pressures sensing tests of a device packaged at atmosphere were performed under a series of vacuum levels (Figure 8b, Video S4). *P*_0_ is the atmospheric pressure (101 kPa) of our laboratory (Suzhou, China). If we turn on the pump, the vacuum levels of vacuum chamber can be changed and hold at random values such as 95, 89, 80, 73, 61 kPa defined as *P*_1_, *P*_2_, *P*_3_, *P*_4_, *P*_5_. The reason for choosing these vacuum levels is that they approximately coincide to the atmosphere of Xi’an, Hohhot, Kunming, Geermu, Lhasa (Figure 8b inset), which are Chinese cities. Simultaneously, the responses of PS for the continuous change of air pressure (101 to 60 kPa) was recorded (Figure 8c). Figure 8d demonstrates that the device still obeys Ohm’s law even under extremely high pressures. In addition, the air pressure measurement range of PS can be adjusted though tuning the vacuum levels of package stage. For instance, the air pressure measurement range of device packaged at 100 Pa is 6 to 101 kPa (Figure S8), which is one order of magnitude wider than that of device packaged under atmosphere.

## 4. Conclusions

We prepared a piezoresistive pressure sensors with sensitivity of 474.13 kPa^−1^ and response time of 2 μs, which are realized through eliminating the pressure independent lateral bulk transporting resistance and enhancing the elastic modulus of polymer elastomer, respectively. Meanwhile, our sensor have a wide pressure sensing range from 0 Pa to 110 kPa, and low detection limit of 0.6 Pa. Combined these improved performance, we successfully fabricated vibration sensors and air pressure sensors based on our sensors. The vibrations with frequency range from 1 to ~300 Hz as well as the air pressures from 6 to 101 kPa can be detected accurately.

## Supplementary Materials

The following are available online at http://www.mdpi.com/2073-4360/10/6/587/s1.
