# Supplementary material for: High-Performance Pressure Sensor for Monitoring Mechanical Vibration and Air Pressure
Source: Polymers (Basel). 2018 May 27;10(6):587. doi: 10.3390/polym10060587 (PMC6403672; doi:10.3390/polym10060587)
Supplement: Supplementary file 1 [file polymers-10-00587-s001.zip › Figure S1-S8.docx]

Supporting Information

**High-Performance Pressure Sensor for Monitoring Mechanical Vibration and Air Pressure**

**Yancheng Meng ^1,2^, Hongwei Li ^2^, KunjieWu ^2^, Suna Zhang ^2^ and Liqiang Li ^1,2,^***

^1^ School of Nano-Technology and Nano-Bionics, University of Science and Technology of China, Hefei 230026, China; [ycmeng2015@sinano.ac.cn](mailto:ycmeng2015@sinano.ac.cn)

^2^ Suzhou Institute of Nano-Tech and Nano-Bionics (SINANO), Chinese Academy of Sciences, Suzhou 215123, China; [hwli2015@sinano.ac.cn](mailto:hwli2015@sinano.ac.cn) (H.L.); [kjwu2014@sinano.ac.cn](mailto:kjwu2014@sinano.ac.cn) (K.W.); snzhang2014@sinano.ac.cn (S.Z.)

***** Correspondence: lqli2014@sinano.ac.cn; Tel.: +86-512-6287-2829

**Figure S1.** The dependency of sensitivity on pressure in high pressure region (5 to 110 kPa).

**Figure S2.** The current increased or decreased and hold with pressure increasing or decreasing and holding in various values for a comparative device.

**Figure S3.** Repeatability test under the load switching between 0 and 14.27 Pa. Ultra-small object, such as silicon slices with the weight of 64.09 mg corresponding to a pressure of only 14.27 Pa, was loaded and removed successively. The current response and relaxation shown regularly and steadily, and the change of which approaching to three orders of magnitude.

Discussion S1. the Calculation Process of Total Resistance of the Sensor

For simplicity purposes, the pyramid-like microstructure was approximated as cone.


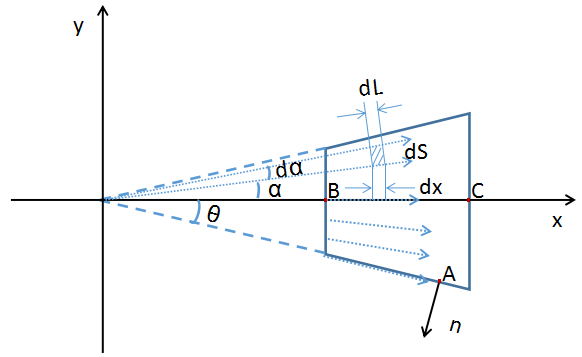


**Figure S4.** The distribution of current in the cone based on the steady electric field approximation. *A* is random point of cone’s surface. *B* and *C* are intersections of x-axis and top and bottom surface of cone, respectively. *θ* is an angle between x-axis and tangent of cone’s surface, *α* is random angle between x-axis and random line across the cone, *dα* is increment of *α*. *dL* and *dS* are thickness and cross sectional area of random slice related to *dα*, respectively. *n* is normal of cone’s surface.


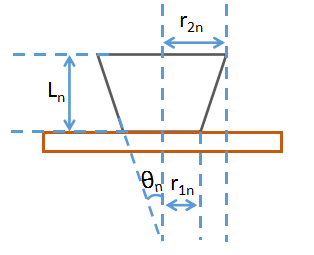


**Figure S5.** The geometry deformation of cone under pressure. *r_1n_* is radius of cone’s section deformed under pressure, *r_2n_* is radius of cone’s back surface (a constant), *L_n_* is a height of cone. With the increasing of pressure, the deformation of cone will be enhanced, resulting in the increase of *r_1n_* and decrease of *L_n_*.

Based on Holm’s theory, contact resistance of cone can be shown as Equation S1

 (S1)

Based on steady electric field, there is Equation S2 at point *A* (Figure S4 Supporting Information)

 (S2)

*n* is normal of cone’s surface, *u* is electric potential of random point of cone. And then the resistance of random slice related to *dS* can be expressed as the following

 (S3)

The resistance of portion between *α* and *α+dα* is *dR*, which can be expressed as Equation S4

 (S4)

By solving Equation S4, the bulk resistance (*R_nbulk_*) of cone can be written as

 (S5)

*r_1n_, r_2n_, L_n_, θ_n_* defined at Supporting Information Figure S5, *ρ* is conductivity of conductive layers.

As analyzed in section of Sensor fabrication and Working Principle (main text), the total resistance of individual pyramid-like microstructure (approximated as cone) can be divided into vertical bulk resistance (*R_nbulk_*, the bulk resistance of cone), contact resistance (*R_ncon_*), bottom electrode resistance (*R_nbottom_*) and top electrode resistance (*R_ntop_*) which are tandem connected.

 (S6)

Because the top and bottom electrodes are highly conductivity Au film and ITO film, respectively, *R_nbottom_* and *R_ntop_* can be neglected. Equation S6 can be reduced to Equation S7

 (S7)

Based on Supporting Information Figure S5, we have following relation

 (S8)

And then

 (S9)

C and D are constant, which are the abbreviations of the product of several constants.

*R_ntotal_* is total resistance of random individual pyramid-like microstructure. There are a large number (*N*) of pyramid-like microstructure below the Au film. Because of the existence of Au film, the resistance of each pyramid-like microstructure can be expressed as Equation S9, and they are connected in parallel mode (main text Figure 1e). So the total resistance (*R_ntotal_*) of the sensor can be expressed as Equation S10

 (S10)

**Figure S6.** Wrist pulse detection. a) The responses of the sensor on the wrist pulse, which show the frequency of 1Hz. b) Random individual pulse waveform. It exhibits three clearly distinguishable wave defined as P_1_, P_2_, P_3_, respectively. P_1_ wave defined as systolic peak is caused by systolic pressure wave that results from blood ejected from the left cardiac chamber to aorta, and its consequent distribution to peripheral sites. P_2_ wave defined as diastolic peak is formed by pressure wave reflected back to the aorta, and P_3_ wave relate to the heart rate.

**Figure S7.** The vibrations with different frequencies were detected by our sensors. To test the generality of our sensor for different machines, the other vibrations with the frequency 50 Hz provided by another pump a), and 25 Hz b), 220 Hz c) provided by mixer were detected. d) Real-time *V-t* curves of the sensor based on the pump vibration with the frequency of 50 Hz. There are 1000 cycles in 20 seconds. The sensor maintained good performance after extremely fast multi-times stimulus.

**Figure S8.** The responses of device packaged at vacuum degree of 100 Pa for the air pressure changes. Its air pressure measurement range is 6 to 101 kPa, which widen one order of magnitude than that of device packaged under atmosphere.
